# Stunned Into Silence: Classical Catatonia secondary to Viral Myocarditis, a Case Report

**DOI:** 10.1192/j.eurpsy.2025.1195

**Published:** 2025-08-26

**Authors:** F. R. Bhatti, S. Liaqat

**Affiliations:** 1 Psychiatry, Pakistan Institute of Medical Sciences, Islamabad, Pakistan; 2 Psychiatry, RUSH University Medical Center, Chicago, United States

## Abstract

**Introduction:**

Catatonia due to myocarditis is rare and poses a significant diagnostic challenge. We present a case of classical catatonia secondary to viral myocarditis.

**Objectives:**

We want to highlight the clinical issues in diagnosing catatonia due to specific medical conditions, especially in a resource-limited setting like Pakistan.

**Methods:**

The case is discussed, followed by suggestions and a literature review.

**Results:**

A 22-year-old male with no known medical and psychiatric comorbidities presented to the emergency department in April 2023 with avolition, alogia and fever for 2 weeks. Patient was admitted to Neurology for workup; CSF analysis, CSF autoimmune screen, CSF culture, CSF HSV PCR, blood culture, urine culture, serum ceruloplasmin, MRI Brain were all unremarkable. He was treated with broad-spectrum antibiotics, following which his fever remitted.

On initial psychiatric exam, patient scored 13 on Bush Francis Catatonia Rating Scale for mutism, staring, posturing/catalepsy, stupor, grimacing, mitgehen, gegenhalten and rigidity. Initial work up included normal CBC, CMP, TSH, HIV/Syphilis, Urine toxicology, ESR and a CRP of 43 mg/L. He underwent echocardiography which revealed a severely reduced Ejection Fraction of 30% and dilated cardiomyopathy, consistent with viral myocarditis; which was considered in remission and thus managed conservatively by Cardiology. The patient then received IV infusion of Diazepam 10mg BID which adequately treated his catatonia over 3 days and he was discharged with instructions for close follow-up. On followup visits, the patient continued to display remission of catatonic symptoms, but exhibited new symptoms of acute psychosis including 2nd person auditory hallucinations and delusions of reference and persecution. These were treated with Olanzapine 5mg PO resulting in complete remission over 4 weeks. The patient continued to have residual symptoms of social withdrawal, decreased motivation and poor concentration on his most recent presentation. Patient was also noted to have an exaggerated startle reflex which was not associated with any other neurologically relevant symptoms. It prompted a workup to rule out seizure disorder, the results for which are pending at time of this submission.

**Conclusions:**

This case underscores the clinical complexity and diagnostic challenge of catatonia linked to medical conditions. It also depicts the evolving presentation over time. It is important to note that the patient did not receive IV Lorazepam due to its unavailability in Pakistan. Electroconvulsive therapy should have been considered for his catatonic symptoms but was delayed due to his pending diagnosis. Limited findings from literature review related to catatonia associated with viral myocarditis demonstrate the need for further research to bridge the gaps in evaluation and management.

**Disclosure of Interest:**

None Declared

